# Biotechnological Control of Hydrogel Properties via Recombinant Protein Molecular Weight Engineering

**DOI:** 10.1002/mabi.202500575

**Published:** 2026-01-28

**Authors:** Domenic Schlauch, Jan Peter Ebbecke, Amelie Paula von Alwörden, Dörte Solle, Selin Kara, Antonina Lavrentieva, Iliyana Pepelanova

**Affiliations:** ^1^ Leibniz University Hannover Hannover DE Germany

**Keywords:** design of experiments, GelMA, hydrogel, molecular weight, recombinant gelatin

## Abstract

Hydrogels based on natural polymers are widely used in 3D cell culture and tissue engineering due to their biocompatibility and tunability. In this work, recombinant collagen‐derived proteins of defined molecular weights were designed and tested as precursors for methacrylated, photocrosslinkable hydrogels. Proteins of 25.6 kDa, 58 kDa, and 89.2 kDa were recombinantly expressed in Komagataella phaffii, methacrylated, and photocrosslinked to form well‐defined hydrogels. A Design of Experiments (DoE) strategy was employed to quantify the effects of degree of functionalization (DoF) and precursor molecular weight on hydrogel stiffness, deformability, and swelling. For the first time, it was reported that both the DoF and molecular weight of recombinant proteins used for hydrogel fabrication significantly influence hydrogel properties. The molecular weight effects were most pronounced at lower chain lengths. Predictive models generated from the DoE revealed non‐linear and interactive contributions of both parameters, while mixed‐material formulations suggested non‐additive behavior beyond the fitted design space. Additionally, biocompatibility for all materials was shown by live‐dead staining of cells seeded onto the crosslinked materials. The results demonstrate that recombinant protein chain length can be used as a powerful design parameter to modulate hydrogel mechanics. Such materials not only enable xeno‐free cultivation but also provide a biotechnological route toward rationally engineered biomaterials for diverse applications.

## Graphical abstract

In this study, the authors described the expression of human collagen‐derived proteins with different molecular weights in *Komagataella phaffii*. The expressed proteins were used for crosslinked hydrogel preparation, and a DoE study showed that the degree of functionalization and molecular weight influenced hydrogel stiffness, swelling, as well as deformability of the gels.

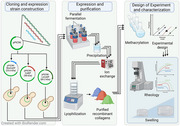

**Abbreviations**
ColDP: Collagen‐derived protein
DoE: Design of Experiments
DO: Dissolved oxygen
DoF: Degree of functionalization
GelMA: Gelatin methacryloyl
LAP: Lithium‐phenyl‐2,4,6‐trimethylbenzoylphosphinate
LoCoDP: Long collagen‐derived protein
MAA: Methacrylic acid anhydride
NMR: Nuclear magnetic resonance
OD_600_
: Optical density at 600 nm
SDS‐PAGE: Sodiumdodecylsulfate acrylamidelectrophoresis
ShoCoDP: Short collagen‐derived protein
TNBS: 2,4,6‐Trinitrobenzene sulfonic acid

## 1 Introduction

Hydrogels, 3D networks of water‐absorbing polymers, are widely used for tissue engineering and medical applications due to their ability to provide a physiological 3D microenvironment for cell growth. They can be produced from a wide variety of materials, including synthetic and natural materials. Hydrogels based on proteins provide important biological functionalities; however, they commonly require the introduction of additional synthetic functional groups in order to introduce more control over mechanical properties and to enable crosslinking in physiological conditions.

Among photocrosslinkable hydrogels, gelatin methacrylate (GelMA) is one of the most widely used materials in 3D cell culture and tissue engineering. It has been applied to the creation of diverse tissue models, including skin [1], muscle [2], and bone [3], and has been adapted to support a broad range of cell types and mechanical properties. Beyond tissue engineering, GelMA also shows promise for clinical applications, for example, as a surgical sealant [4]. Key advantages of GelMA include its simple synthesis, broad availability, intrinsic cell‐adhesive properties [5], and biodegradability in vivo, which together support tissue remodeling and enable vascularization upon implantation [6].

GelMA is prepared by functionalization of gelatin with methacryloyl groups, resulting in a material that retains the gelatin's favorable biological properties, while being able to undergo rapid crosslinking in the presence of a photoinitiator and light [7], making it easy to fabricate 3D matrices with it.

It has been shown that mechanical cues influence cell growth [8] and differentiation [9, 10], so the precise control over mechanical properties is highly desirable. In GelMA hydrogels, control over the mechanical properties of the material is mainly achieved by adjusting several parameters. The pre‐polymer concentration [7, 11], and the light dosage during crosslinking [2, 11] can be controlled during hydrogel preparation. Furthermore, the degree of functionalization (DoF) can be varied to influence hydrogel properties [7, 11, 12], while DoF itself can be adjusted by changing the relative educt ratio compared to the material mass during material functionalization [13]. While increasing these parameters effectively boosts material stiffness, other related hydrogel properties, such as swelling [12], and the effects on cell spreading, migration [14], and proliferation [15] are also affected. As a result, the desired hydrogel properties need to be carefully tuned by variation of the above‐mentioned factors. However, while one hydrogel as property can be adjusted, other attributes are inevitably affected by the change of these parameters as well. For example, an increase in stiffness resulting from an increase in DoF will also change the material's swelling. Furthermore, the levels to which DoF, crosslinking time, and pre‐polymer concentration can be adjusted are limited. This limitation stems from parameter settings where the material is able to effectively form a hydrogel by sufficient crosslinking or by reaching a physical limitation, such as maximum DoF achievable or a limit in solubility. Therefore, to gain more flexibility in the design of hydrogels with specific properties, additional ways of influencing the mechanical behavior are desirable.

Another way of influencing the mechanical properties of hydrogels is opened up by the choice of the pre‐polymer itself. While a change of pre‐polymer parameters is relatively easy for synthetic materials, it is more challenging for biological polymers like gelatin, as their extraction processes are usually more complex and often require harsh conditions. As a result, the obtained materials are present as a mixture of different molecular weights [16]. Here, the use of recombinant proteins of a certain length as hydrogel materials opens up a promising way of tailoring hydrogel properties, as their composition can be precisely controlled by rational design.

Recently, we reported the fabrication of a hydrogel based on recombinantly produced collagen fragments. In this previous study, a recombinant collagen‐derived protein was functionalized with methacryloyl groups, and the formed hydrogel was compared to GelMA as a standard material in 3D cell culture. The recombinant material described there mimicked GelMA in most of its properties, while lacking its thermal gel formation at ambient temperatures, thereby simplifying its handling [17]. In the here‐presented study, we aimed to explore whether the protein chain length could serve as an additional parameter to modulate the mechanical properties of the resulting hydrogel. For this, collagen fragments of three different chain lengths were recombinantly expressed in the methylotrophic yeast *Komagataella phaffii*. The obtained proteins were first analyzed for their native thermal gelation behavior. They were then modified by methacrylation, yielding recombinant GelMAs of defined molecular weights. A design of experiments (DoE) was carried out, characterizing the influence of the degree of functionalization and protein molecular weight on mechanical properties and hydrogel swelling. Our study demonstrates a predictive model relating protein molecular weight and DoF to the resulting specific mechanical properties of hydrogel constructs.

## 2 Material and Methods

All chemicals were purchased from Carl Roth unless otherwise stated. Restriction enzymes were purchased from Thermo Fisher Scientific.

### 2.1 Cloning and Generation of Expression Strains

The middle‐sized collagen fragment (ColDP) was obtained from a previous study [17]. In short, the coding sequence of a 58 kDa fragment of the human alpha‐1 type I collagen was ordered from Invitrogen. The product was codon optimized for expression in *Komagataella phaffii*. The gene synthesis product was cut using Fast Digest PsiI and NcoI and ligated into the pPIC9K vector (Invitrogen), cut with the same restriction enzymes. The resulting plasmid was then transformed into Top10 *E. coli* (Invitrogen).

For the long collagen‐derived protein (LoCoDP), a part of the coding sequence of ColDP was copied by PCR and added again into the ColDP coding sequence by homologous recombination to increase the molecular weight of the expressed protein. The method used was published by Beyer et al. [18] and was used because of its scar‐free cloning result in the open reading frame of the desired protein. For this, a part of the collagen coding sequence was amplified using the primers 5’‐gacctcctggaccaccaaaaggtgatagaggtgatgc‐3’ and 5’‐aattcgcggccgctattacttatcgcctctgggacc ‐3’ by PCR, while the remaining pPIC9K‐ColDP plasmid was amplified using the primers 5’‐cccagaggcgataagtaatagcggccgcgaattaatt‐3’ and 5’‐acctctatcaccttttggtggtccaggaggtcc‐3’. By this, homologous overlapping regions were introduced in the ColDP coding sequences, and the amplified piece of the collagen coding sequence could be added by homologous recombination following transformation into Top10 *E. coli*, increasing the molecular weight of the collagen expressed from 58 kDa to 89.2 kDa.

The short collagen‐derived protein (ShoCoDP) with 25.6 kDa molecular weight was generated by amplifying a part of the ColDP sequence using the primers 5’‐gactggttccaattgacaagc‐3’ and 5‘‐attcgcggccgctattatttagcacctggttgtccatc‐3‘. The PCR product as well as the pPIC9K vector, were cut using Fast Digest EcoRI and NotI and ligated before being transformed into Top10 *E. coli*.

The coding sequences of all expression constructs were verified by Sanger sequencing using the LightRun service (Eurofins Genomics, Germany).

For the generation of stable expression strains, the plasmids were linearized using Fast Digest SalI before being introduced into *K. phaffii* by electroporation. Transformants were spread onto selective agar plates without histidine and incubated at 30 C for 3 days. Transformants grown on the plate were then pooled and plated in YPD agar plates with increasing geneticin concentrations to screen for clones bearing multiple integrations. Several clones showing geneticin resistance were then chosen for expression in shake flask experiments, and suitable clones were selected for further expression experiments of the proteins.

The protein sequences of all recombinant collagens are given in the Supporting Information.

### 2.2 Expression and Purification of Recombinant Collagen Fragments

The seed culture was prepared by inoculating 50 mL of YPD medium (10 g L^−1^ yeast extract, 20 g L^−1^ tryptone, and 10 g L^−1^ glucose) from a frozen stock in a 250 mL baffled Erlenmeyer flask. The pre‐culture was incubated at 30 °C and 150 rpm for 16 h. High cell density fermentation was performed in a DASGIP parallel bioreactor system (Eppendorf, Germany) in a fed‐batch process as described in a pre‐print before [19]. For this, 700 mL of medium (60 g L^−1^ glycerol, 9 g L^−1^ ammonium sulfate, 11.67 g L^−1^ magnesium sulfate [Merck] 0.9 g L^−1^ calcium sulfate dihydrate [VWR], 14.67 g L^−1^ potassium sulfate, 25.05 g L^−1^ sodium hexametaphosphate [Sigma‐Aldrich], 200 µL L^−1^ Tego KS911 antifoam [Evonik] and 3.35 mL L^−1^ PTM1 trace salt solution [VWR]) were prepared in each of the vessels. The reactors were equipped with temperature, pH, and dissolved oxygen (DO) sensors. The pH was controlled by the addition of 3 m HCl and 25% ammonia. The reactors were inoculated from the pre‐culture to an optical density (OD_600_) of 1, and the fermentation was carried out at pH 5 and 30 °C and a gas flow of 50 sL h^−1^. The DO was maintained at a minimum of 30% by increasing the stirring speed and supplementing oxygen to the gas if needed. After complete consumption of glycerol, seen by a sudden spike in DO signal, a methanol feed (methanol with 12 mL L^−1^ of PTM1 trace salt) was started with a flow rate of 3.6 mL h^−1^ for 4 h. The methanol feed was then increased to 7.3 mL h^−1^ for 2 h before it was set to 10.9 mL h^−1^ for the rest of the fermentation. The process was ended after 112 h.

The fermentation broth was centrifuged at 4400 g for 30 min. Remaining cells were removed by filtration with a 0.2 µm bottletop filter. The filtrate was then dialyzed against H_2_O by tangential flow filtration on a Sartoflow Smart (Sartorius, Germany) with a 10 kDa cutoff membrane installed. The dialysis was performed until a steady conductivity was reached. The retentate was then lyophilized for storage. The dry product was dissolved in H_2_O at 20 mg mL^−1^ before ammonium sulfate was added at 30% of its solubility. The protein was allowed to precipitate for 1 h at 4 °C and then centrifuged at 4400 g for 1 h. The pellet was resuspended in H_2_O and dialyzed against H_2_O for 16 h using dialysis tubes with a molecular cutoff weight of 14 kDa (Spectra Por) to remove remaining ammonium sulfate. The precipitated protein was then lyophilized for storage. Following precipitation, the protein was dissolved in TRIS buffer (0.2 m, pH 7.2) at a concentration of 20 mg mL^−1^. The protein was then purified using a 5 mL HiTrap Q HP column (Cytiva, Sweden), where the recombinant collagen was collected as flow‐through during the sample loading. Impurities bound to the column were then eluted using 0.2 m TRIS buffer at pH 7.2 containing 0.2 m NaCl. The collected flow through was again dialyzed against H_2_O using a MWCO of 14 kDa and lyophilized to obtain a dry product.

### 2.3 SDS‐PAGE

For analysis by SDS‐PAGE, 30 µL of each sample was mixed with 10 µL Laemmli buffer (4x) and incubated at 96 °C for 5 min. Per sample, 8 µL were loaded onto a 12 % gel. For size comparison, a pre‐stained protein marker (Thermo Fisher Scientific) was added. Proteins in the gel were stained with Coomassie Brilliant Blue G 250 by overnight incubation.

### 2.4 Experimental Design

A Design of Experiment study was performed for material characterization, where different educt ratios resulting in different DoF and the molecular weight of the protein were used as influencing factors. In total, 9 experiments were performed varying these factors. ShoCoDP and LoCoDP were analyzed at a low and high DoF, while ColDP was analyzed at an intermediate DoF. The resulting hydrogel's stiffness, swelling, and yield point were analyzed. Evaluation of the DoE was performed using the MODDE software (Version 13.1, Sartorius, Germany). In the DoE evaluation educt ratio was exchanged for DoF, as the educt ratio and resulting DoF are correlated, and DoF is typically used in the literature as the defining parameter for materials.

### 2.5 Functionalization

The recombinant collagen‐derived proteins were functionalized by reaction with methacrylic anhydride based on the protocol published by Shirahama & Lee [13]. For this, the proteins were dissolved at a concentration of 10% (w/v) in 0.25 m carbonate‐bicarbonate (CB) buffer at pH 9 and 30°C. After dissolving, the pH was adjusted to 9 using 1 m NaOH. Subsequently, methacrylic acid anhydride (MAA) was dropwise added to the reaction in different ratios according to the design space determined by the DoE (0.3 mL g^−1^, 0.35 mL g^−1^ and 0.1 mL g^−1^ for ShoCoDP; 0.45 mL g^−1^ and 0.6 mL g^−1^ for ColDP and 0.3 mL g^−1^ and 0.1 mL g^−1^ for LoCoDP). The reaction was allowed to proceed for 3 h, before it was terminated by adjusting the pH to 7 using 1 m HCl. The reaction mixture was filled into dialysis tubes with a MWCO of 14 kDa and dialyzed against H_2_O for 20 h. The functionalized protein was freeze‐dried for storage. An overview of the materials synthesized with the molecular weight and DoF is given in Table 1.

**TABLE 1:** Overview of the samples with the corresponding molecular weight, educt ratio, and DoF.

Material name	Short name	Molecular weight [kDa]	MAA/Protein [mL g^−1^]	DoF [%]
ShoCoDP‐MA Low	SL	25.6	0.035	60
ShoCoDP‐MA High	SH	25.6	0.1	77.66
LoCoDP‐MA Low	LL	89.2	0.03	41.98
LoCoDP‐MA High	LH	89.2	0.1	87.52
ColDP‐MA Mid 1	—	58	0.6	81.8
ColDP‐MA Mid 2	—	58	0.45	58.3
ColDP‐MA Mid 3	—	58	0.45	58

### 2.6 TNBS Assay

For the determination of the DoF, the primary amines of non‐functionalized and functionalized recombinant collagen‐derived proteins were reacted with 2,4,6‐trinitrobenzenesulfonic acid (TBNS) as described by Lee & Shirahama [20]. Briefly, 1.6 mg mL^−1^ functionalized and 0.4 mg mL^−1^ non‐functionalized protein were dissolved in 500 µL of 0.1 m CB buffer each, and 500 µL of 0.01% TNBS in 0.1 m CB buffer was added. The reaction was allowed for 2 h at 37°C before it was stopped by the addition of 500 µL 10% SDS and 250 µL 1 m HCl. The absorbance of the samples was then measured at 335 nm. Reactions were performed in triplicate. For quantification of the primary amine content, the absorbance values were compared to a glycine standard containing 0 mg mL^−1^, 1.25 mg mL^−1^, 2.5 mg mL^−1^, 5 mg mL^−1^, 10 mg mL^−1^, and 20 mg mL^−1^. The DoF was then calculated as the difference in primary amines in the functionalized and non‐functionalized protein divided by the primary amine content in the non‐functionalized protein.

### 2.7 Nuclear Magnetic Resonance (NMR) Spectroscopy

For NMR analysis of functionalization, 20 mg of unfunctionalized and functionalized collagen were dissolved in 1 mL D_2_O. ^1^H NMR spectra were recorded on an ASC600 (Bruker, USA). NMR spectra were analyzed using the MestReNova software (MestReLab Research, Spain).

### 2.8 Rheology

For thermal gelation experiments, all proteins were dissolved at 10% (w/v) in H_2_O. Storage modulus was measured on an Anton Paar MCR302 rheometer (Anton Paar, Germany) at a constant deformation of 0.1% and 1Hz. The temperature was decreased from 37°C to 4 C with a cooling rate of 1°C min^−1^. After reaching 4°C, the temperature was increased again to 37°C at the same rate.

For crosslinking experiments, the functionalized proteins were dissolved at 10% (w/v) in H_2_O containing 0.1% (w/v) lithium‐phenyl‐2,4,6‐trimethylbenzoylphosphinate (LAP). Storage and loss modulus were measured at 30°C at 0.1% deformation and 1 Hz. During the measurement, the materials were irradiated with UV light at 365 nm with 10 mW cm^−2^ for 200 s.

Amplitude sweep experiments were performed by polymerizing the material at 7.5% (w/v) with 0.1% (w/v) LAP in H_2_O for 300 s without disturbance to ensure crosslinking. The amplitude was then increased from 0.1% to 2000% while storage and loss modulus were recorded.

### 2.9 Hydrogel Swelling

For swelling experiments, hydrogels were prepared by dissolving the functionalized proteins in H_2_O at 10% (w/v), containing 0.1% (w/v) LAP. In contrast to crosslinking in situ on the rheometer, the solution was cast into molds, and the gels were cured with 1.2 J cm^−2^ in a Vilber Biolinker UV chamber (Vilber, Germany), which was sufficient to form stable hydrogels for all materials. The gels were then removed from the molds and soaked in excess volume of H_2_O for 24 h. The swollen gels were then weighed before being lyophilized, and the dry gels were weighed again. The swelling ratio was calculated by the fraction of the swollen weight and the dry weight of each gel.

### 2.10 Cell Culture

Human adipose tissue‐derived mesenchymal stem/stromal cells (hAD‐MSCs) were obtained from previous work [21]. All patients provided their informed consent, as approved by the Institutional Review Board (Hannover Medical School) with the reference number 3475‐2017. Cells were cultured in alpha‐MEM (Gibco, Thermo Fisher Scientific, Germany) containing 10% human serum (c.c.Pro, Germany) and 0.05 mg mL‐1 gentamycin (Merck KGaA). Recombinant methacrylated collagen‐derived proteins were dissolved at 10% (w/v) in RPMI containing 0.1% LAP and sterile filtered. Hydrogels were formed by pipetting 50 µL of the solution per well in a 96‐well flat‐bottom plate (Sarstedt, Germany) and subsequent crosslinking in a Vilber Biolinker with 1.2 J cm^−2^. 5000 hAD‐MSCs were seeded per well in 200 µL alpha‐MEM. Cells were incubated at 37°C and 5% CO_2_. After three days, live/dead staining was performed using 7 µm Calcein‐AM and 1.5 µg mL^−1^ propidium iodide. The cells were imaged using an IncuCyte S3 microscope (Sartorius, Germany)

## 3 Results and Discussion

### 3.1 Expression of Recombinant Collagen‐Derived Proteins with Different Molecular Weight

**FIGURE 1:**
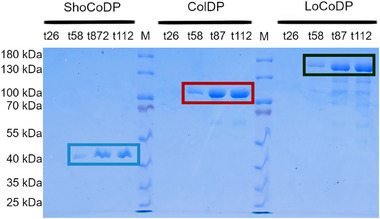
SDS‐PAGE of the expressed collagen‐derived proteins of different molecular weights. ShoCoDP is seen above 40 kDa (blue box), ColDP above 100 kDa (red box), and LoCoDP between 130 kDa and 180 kDa (green box).

Collagen‐derived proteins with a molecular weight of 25.6 kDa, 58 kDa, and 89.2 kDa were recombinantly expressed in *K. phaffii* and secreted in the culture supernatant. An overview of the data recorded during the fermentation is given in the in Figure S1. Protein secretion to the culture supernatant was analyzed by SDS‐PAGE as shown in Figure 1. Following induction after 26 h, protein bands of increasing intensity were observed at 40 kDa for ShoCoDP, at 100 kDa for ColDP, and between 130 kDa and 180 kDa for LoCoDP. The observed molecular weights were higher than the actual molecular weights of the protein. This abnormal migration pattern has been described before for recombinant collagens [22, 23].

In ColDP and LoCoDP, less intense bands at lower molecular weights were also observed. These bands are most likely to arise from fragments of the expressed protein, as the fragmentation of similar recombinant collagens has been described before [23], probably resulting from proteolytic degradation at specific sequence motifs [24]. The absence of these bands for ShoCoDP might arise from its shorter polypeptide chain, making degradation less probable and possibly lacking the recognition motifs for proteolytic cleavage.

### 3.2 Thermal Gelation Properties of Recombinant Collagens of Different Molecular Weight

**FIGURE 2:**
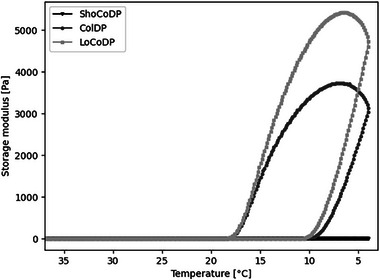
Thermal gelation of ShoCoDP, ColDP, and LoCoDP at 10% (w/v) in H_2_O. Storage modulus was measured at a constant deformation of 0.1% and 1 Hz with a constant cooling/heating rate of 1°C min^−1^ between 37°C and 4°C.

Recombinant collagen‐derived proteins were expressed in *K. phaffii* without co‐expression of the prolyl‐4‐hydroxylase enzyme complex. This results in recombinant collagens without the hydroxylation pattern responsible for the thermal gel formation observed in gelatin. Consequently, the recombinant gelatin showed no thermal gelation at room temperature, simplifying the handling procedure of the material in a previous study [17]. To understand how variation in the protein molecular weight influences the thermal gelation, the gelling of the different protein pre‐polymers was investigated. For this, the storage modulus of the proteins was measured during temperature shift by rheology. As shown in Figure 2, ShoCoDP did not demonstrate any thermal gel formation at the measured concentration and temperature range. In contrast, ColDP and LoCoDP both exhibited a rapid increase in storage modulus at 10°C, with LoCoDP showing a slightly higher gelation temperature and a steeper increase in storage modulus compared to ColDP. The storage modulus following thermal gelation was also higher for LoCoDP. Both materials exhibited a decrease in storage modulus when the sample temperature was increased, again showing that gel formation is reversible.

These results indicate that molecular weight does, in fact, influence thermal gelation. There seems to be a threshold level of minimum molecular weight to undergo thermal gelation for recombinant non‐hydroxylated collagens. Furthermore, higher molecular weight led to stiffer gels in the recombinant collagens. Gelatin with a higher bloom number has been shown to gel at higher temperatures [16, 25]. A higher bloom number stems from a higher average protein chain length and, therefore higher average molecular weight [16]. In recombinant collagens, the difference in gelation temperature was less pronounced compared to gelatin, which probably results from a different gelation mechanism not based on triple‐helix formation, as shown in a previous study [17], but rather in chain entanglement or other intermolecular forces, such as van‐der‐Waals or hydrogen bonding between the protein backbone. Here, longer protein chains provide more possibilities for these types of interactions, which could explain the observed differences in gelation behavior.

### 3.3 Functionalization and DoE Results

The recombinant collagen chains were methacrylated to generate photoactive recombinant hydrogel materials of defined chain length. A DoE strategy was applied to systematically map the design space, quantifying the individual and interactive effects of the DoF and precursor molecular weight on the resulting hydrogel properties.

**FIGURE 3:**
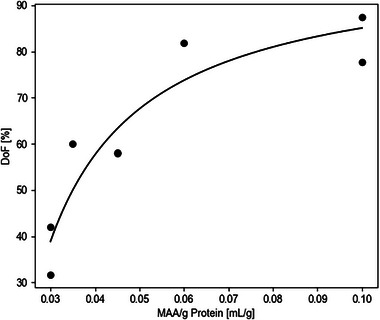
Methacrylic anhydride amount used in the synthesis reaction and resulting degree of functionalization for the materials prepared in this study.

Collagens of different molecular weights were functionalized with different amounts of MAA per gram protein. The MAA ratios chosen were 0.03 mL g^−1^, 0.035 mL g^−1^, and 0.1 mL g^−1^ for ShoCoDP, 0.045 mL g^−1^, and 0.6 mL g^−1^ for ColDP, and 0.03 mL g^−1^ and 0.1 mL g^−1^ for LoCoDP. The adjustments for ShoCoDP from 0.03 mL g^−1^ to 0.035 mL g^−1^, as well as the decrease for ColDP from 0.6 mL g^−1^ to 0.45 mL g^−1^ were made based on initial results to achieve a better distribution of the DoF in the design space. The resulting DoF was then analyzed by TNBS assay and is given in Table 1 and displayed in Figure 3. As seen in Figure 3, the resulting DoF increases with more MAA added per g of protein. The increase in DoF is more pronounced at lower MAA concentrations than at higher ratios [13]. As a result, the DoF can be described by fitting a function of the form $DoF=100\;\times \;\frac{[MAA]-A}{B+([MAA]-\;A)}$ to the data points. Here, [MAA] is the volume of MAA in mL added per gram protein with the parameters A = 0.02122 and B = 0.01379. Because of this direct connection between DoF and MAA ratio during synthesis, the remaining DoE was evaluated by taking DoF and molecular weight as input parameters.

Hydrogel stiffness after UV crosslinking, deformation before breaking of the hydrogels as analyzed by an amplitude sweep, as well as hydrogel mass swelling ratio, were taken as result outputs. All parameters and results are summarized in Table S1.

**FIGURE 4:**
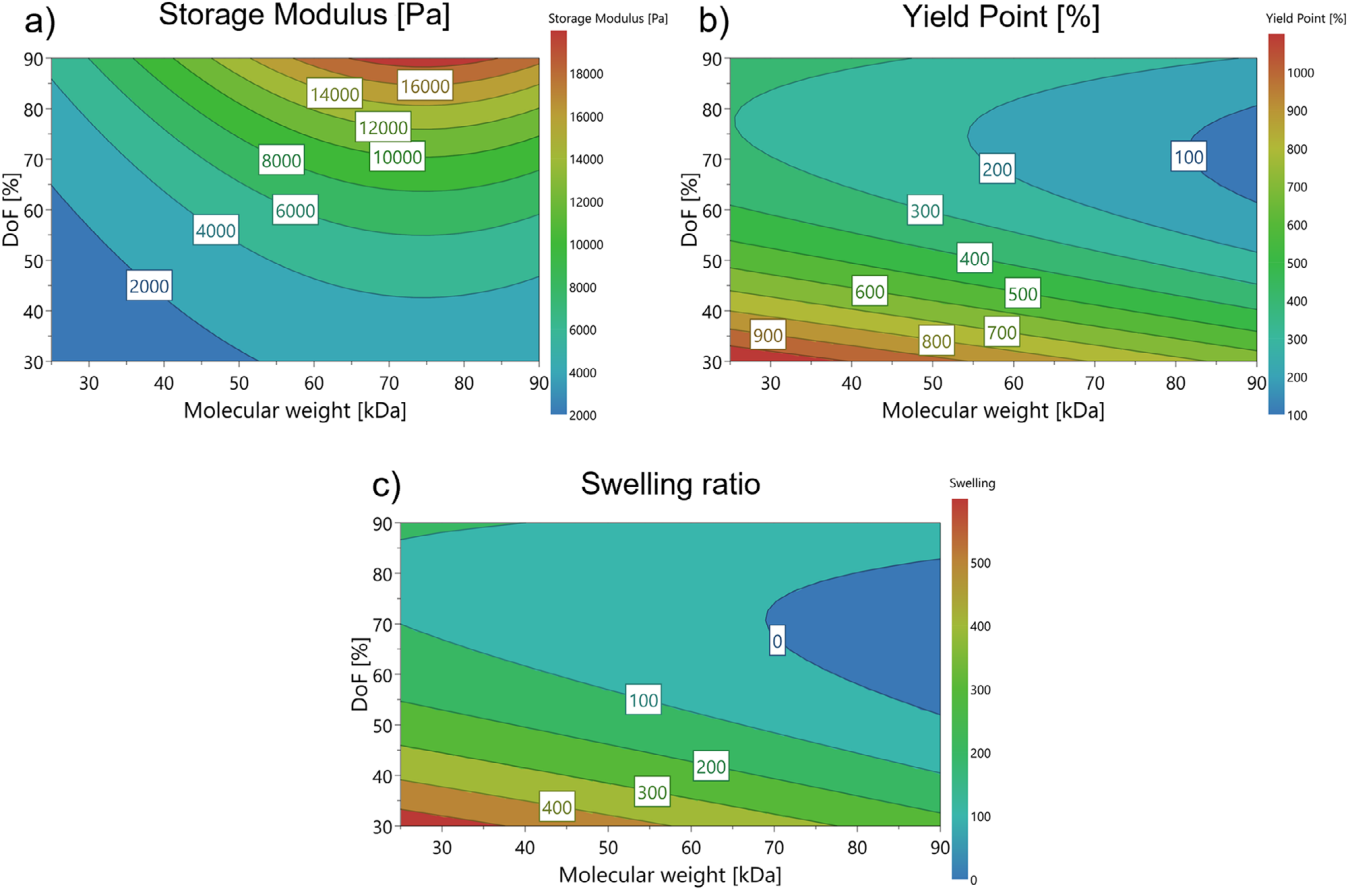
Response contour plots of the recombinant hydrogels as a result of DoF and molecular weight for a) hydrogel stiffness, b) yield point, and c) hydrogel mass swelling ratio.

Our analysis revealed that, for every hydrogel characteristic studied, both DoF and molecular weight significantly contributed to the observed material properties (see Figure 4; Figure S3). The storage modulus was transformed using a decimal logarithm for fitting the model, due to the large distance in the observed stiffness between the lowest/middle and highest measured values.

The DoF showed linear influence on the stiffness after UV‐crosslinking, while molecular weight was found to have a non‐linear effect on the hydrogel stiffness (See Figure 4a; Figure S3a). The influence of molecular weight on hydrogel stiffness was most pronounced for proteins with lower chain length, while the effect diminished for larger proteins. Although the role of DoF in modulating the stiffness of collagen‐based hydrogels is well studied [7, 14], the impact of recombinant protein molecular weight has not been previously reported.

For gelatin‐based materials, a correlation between bloom strength and hydrogel stiffness has been described [25, 26, 27, 28]. The increase in stiffness has been linked to a higher content of triple helices in gels made from gelatin with a higher bloom number [27]. This is also a physical crosslinking component influencing the gel's mechanical properties. In contrast, the recombinant gelatin‐based materials presented in this study do not form triple helices, and no physical crosslinking was observed at the temperatures at which the measurements were performed. Thus, helix formation cannot explain the increase in stiffness after crosslinking for the recombinant materials. Furthermore, UV‐crosslinked GelMA measured at 37°C – above the melting temperature usually observed for the triple helices of gelatin– also showed a correlation between bloom number and hydrogel stiffness [25]. Together with the observation made for the recombinant materials tested, this strengthens the hypothesis that the molecular weight of the protein influences the gel stiffness. Hyaluronic acid methacrylate—despite its similar crosslinking chemistry and overall molecular structure—did not reveal a clear relationship between molecular weight and stiffness [29], likely due to the high viscosity of the precursor solutions, which may interfere with crosslinking efficiency.

For physically crosslinked materials, a correlation between increased stiffness and higher molecular weight has been described for gelatin [30] and alginate [31]. Interestingly, for recombinant collagens with terminal PGP‐repeats that undergo helix formation and, as a result, form a hydrogel upon long incubation times, an increase in molecular weight led to reduced hydrogel stiffness. This behavior results from the decreased protein portion that is involved in the physical crosslinking [32]. Taken together, this suggests that increasing molecular weight alone without an additional part of the molecule being involved in crosslinking does not increase the stiffness of the resulting gel. In contrast to proteins with terminal PGP‐gelation domains, the recombinant proteins described here are crosslinked through photoactive groups on functionalized lysine residues, which are spread throughout the whole protein structure. As a result, an increase in protein molecular weight does not reduce the content of protein involved in crosslinking, as is the case in proteins with terminal PGP‐repeats, and therefore, does not reduce mechanical stability.

For recombinant protein‐based crosslinked materials, a potential link between molecular weight and stiffness has been suggested, but not systematically investigated, where the observed increase in storage modulus for higher molecular weight proteins is thought to result from additional chain entanglements, which reinforce the network independently of crosslinking density [33]. While this study was able to confirm that molecular weight in fact influences hydrogel stiffness, the underlying mechanism of entanglement is still hypothetical and has to be further investigated. Possibly, also the different number of crosslinkable groups per molecule at the same DoF alters network architecture and therefore influences the stiffness of the gels. However, as material availability was limited in this work, the focus was placed on an explorative description of hydrogel properties rather than investigating the distinctive mechanism causing the influence of the molecular weight on hydrogel properties.

Another material property investigated in the recombinant materials was the yield point, or the deformability of the photocrosslinked hydrogels, before the breaking of the molecular structure occurs. Here, DoF showed a non‐linear effect on the deformability, while molecular weight exhibited a linear effect. Furthermore, the model suggests an interactive effect between DoF and molecular weight in regard to deformability (See Figure 4b; Figure S3b). However, the effect of molecular weight was lower compared to DoF, indicating that the elasticity of the material is mainly determined by its crosslinking density.

Another aspect studied in the recombinant hydrogels was their swelling behavior. Here, the same effects were found significant as for the yield point (See Figure 4c; Figure S3c). Again, the effect found for DoF was stronger than the influence of molecular weight on hydrogel swelling.

It remains unclear from the literature whether molecular weight influences the mass swelling ratio. Some studies report that higher bloom number of gelatins led to increased swelling [25] others found no significant effect of bloom number on swelling behavior [26]. In contrast, decreased swelling ratios in hyaluronic acid methacrylate with increasing molecular weight were reported at lower DoF [29]. Furthermore, at high crosslinking degrees, gelatins of different bloom numbers show drastically reduced swelling rates, and differences observable in swelling before crosslinking were absent after the gels were crosslinked [27]. This trend could also be observed in this study. An increase in molecular weight caused a reduced swelling rate at lower DoF. However, as DoF increases, the differences in swelling ratio between the different proteins studied here diminish.

A higher DoF corresponds to lower swelling ratios [34] resulting from the increased crosslinking density of the material. This effect seems to abolish differences in mass swelling ratio stemming from different molecular weights of the hydrogel precursor at higher crosslinking degrees in different types of materials. While in gelatin, the additional physical gelation can influence swelling properties at different bloom numbers, hyaluronic acid, a polysaccharide chain not physically gelling, as well as recombinant non‐hydroxylated collagen, also not showing physical crosslinking, both show a similar influence of molecular weight on swelling at low crosslinking densities.

It has to be noted that while swelling experiments can be directly compared between different materials, the overall hydrogel characteristics might be different compared to the hydrogels prepared during rheological measurements. This possible difference results from the preparation of hydrogels for swelling in molds directly on glass slides to minimize the required physical manipulation of the gels following crosslinking, and therefore the use of an alternative light source.

In conclusion, the DoE showed a clear effect of the molecular weight of the recombinant protein precursor on the resulting hydrogel properties. It has to be noted for all models that, especially the quadratic effects, might not exactly reflect the actual material behavior, but rather model a saturation effect at high values. While the observed data points could be well explained by the model, an increase in swelling and deformation before breaking above 80% DoF is in contrast to the mechanistic explanation of the material's behavior and is most likely attributed to the quadratic fitting function. The generated models could be improved by further expanding the design space and data points for model fitting in the future.

Besides the materials' molecular weight and DoF, the hydrogels properties were also targeted by mixing materials of different molecular weight and DoF to achieve more flexibility in tuning the gels properties. However, due to material limitations, only one replicate measuring the storage modulus for each combination could be measured (See Figure S4). It seems that a mixture of different molecular weights and DoFs could be employed in the future to further target gel characteristics. However, more extensive characterization is necessary for precise characterization of the resulting hydrogels' properties, which was beyond the scope of this study.

**FIGURE 5:**
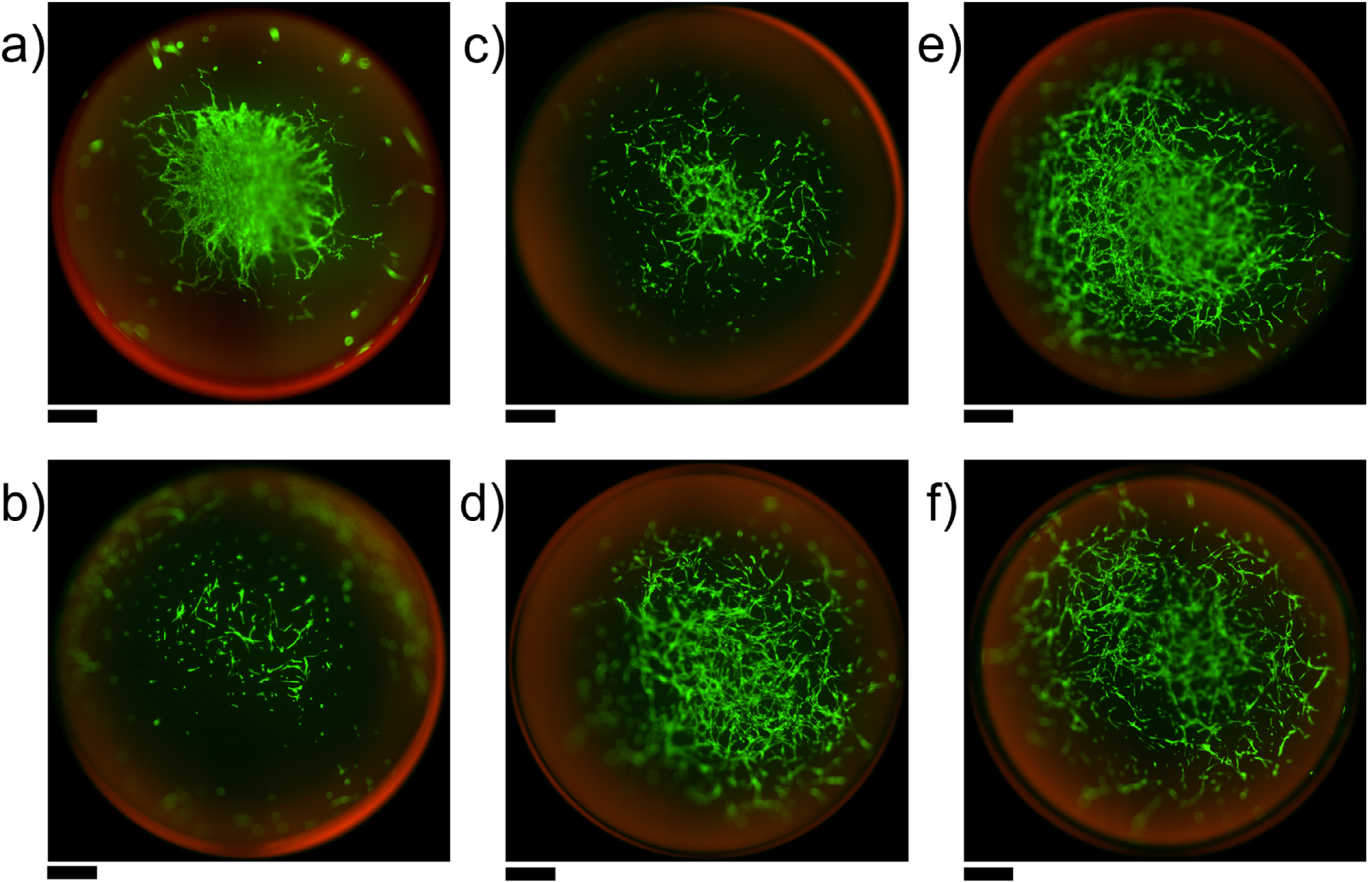
Live dead staining with Calcein‐AM (green) and propidium iodide (red) of human adipose‐derived mesenchymal stromal cells seeded on a) ShoCoDP‐MA with low DoF, b) ShoCoDP‐MA with high DoF, c) ColDP‐MA with medium DoF, and d) ColDP‐MA with high DoF, e) LoCoDP‐MA with low DoF, f) LoCoDP‐MA with high DoF. Cells were cultured for 3 days prior to staining. Scale bars: 800 µm.

Following mechanical characterization, human mesenchymal stromal cells were seeded onto the different materials, and live‐dead staining was performed to showcase the materials’ biocompatibility. As seen in Figure 5, MSCs adhered to all materials, and adherent cells showed spreading and high viability after 3 days of culture. For ShoCoDP with a low DoF (Figure 5a), the cells seemed to aggregate in the center. As this was by far the softest material, this might indicate that cells prefer stiffer substrates. However, as only one replicate was performed due to the scarcity of the material, results should be interpreted with care. Biological replicates are needed in future studies to verify if, in fact, the difference in material stiffness causes aberrant adhesion behavior of MSCs. While the results demonstrate the general biocompatibility of the material, a more in‐depth biological characterization regarding the influence of the different materials on cell spreading, migration, proliferation, and differentiation is desirable in the future.

## 4 Conclusion

This study demonstrates for the first time that by designing the length of recombinant collagen‐derived proteins, it is possible to control the final material properties. It was shown that molecular weight, along with the DoF, is the key determinant of the mechanical and swelling properties of methacrylated, photocrosslinked hydrogels.

Using a Design of Experiments approach, predictive models linking these parameters to stiffness, deformability, and swelling were established, thereby expanding the toolbox for rational hydrogel design. Furthermore, it was proven that changing the protein molecular weight does not adversely influence the improved thermal gelation behavior of the recombinant materials. While limitations in material availability restricted the design space, the findings provide evidence that the chain length of recombinant collagen‐derived proteins influences hydrogel properties.

These results highlight the potential of biotechnology to tailor xeno‐free protein‐based biomaterials at the molecular level, paving the way for more precise design of hydrogels for 3D cell culture and tissue engineering applications. Our future work will be focused on the more in‐depth biological characterization of different recombinant materials regarding their influence on cellular behavior.

## Funding

Funded by zukunft.niedersachsen, a funding program of the Lower Saxony Ministry of Science and Culture and the Volkswagen Foundation as part of the “Matrix Evolution – Hierarchisch strukturierte, bioinspirierte Matrizes” project. Part of this research was financially supported by the German Federal Ministry of Research, Technology, and Space under grant number 13XP5109A.

## Ethics Approval Statement

Primary cells were obtained in accordance with the Institutional Review Board (Hannover Medical School) with the reference number 3475‐2017.

## Patient Consent Statement

All Patients provided their informed consent in accordance to the Review Board decision stated under Ethics approval statement.

## Conflicts of Interest

The authors declare no conflict of interest.

## Supporting information




**Supporting File**: mabi70136‐sup‐0001‐SuppMat.docx.

## Data Availability

The data that support the findings of this study are available from the corresponding author upon reasonable request.
