# Supplementary material for: Biotechnological Control of Hydrogel Properties via Recombinant Protein Molecular Weight Engineering
Source: Macromol Biosci. 2026 Jan 28;26(1):e00575. doi: 10.1002/mabi.202500575 (PMC12852974; doi:10.1002/mabi.202500575)
Supplement: Supplementary file 1 — Supporting File: mabi70136‐sup‐0001‐SuppMat.docx. [file MABI-26-e00575-s001.docx]

Biotechnological Control of Hydrogel Properties via Recombinant Protein Molecular Weight Engineering

Supporting Information (SI)

Domenic Schlauch^[[1]](#footnote-1)^, Jan Peter Ebbecke^1^, Amelie Paula von Alwörden^1^, Dörte Solle^1^, Selin Kara^1^, Antonina Lavrentieva*^1^, Iliyana Pepelanova^1^

^1^ Institute of Technical Chemistry, Leibniz University Hannover, Callinstraße 5, 30167 Hannover, Germany.

^*^ Corresponding author

**E-mail:** [lavrentieva@iftc.uni-hannover.de](mailto:lavrentieva@iftc.uni-hannover.de)

### Online Data and cell growth during fermentation procedures


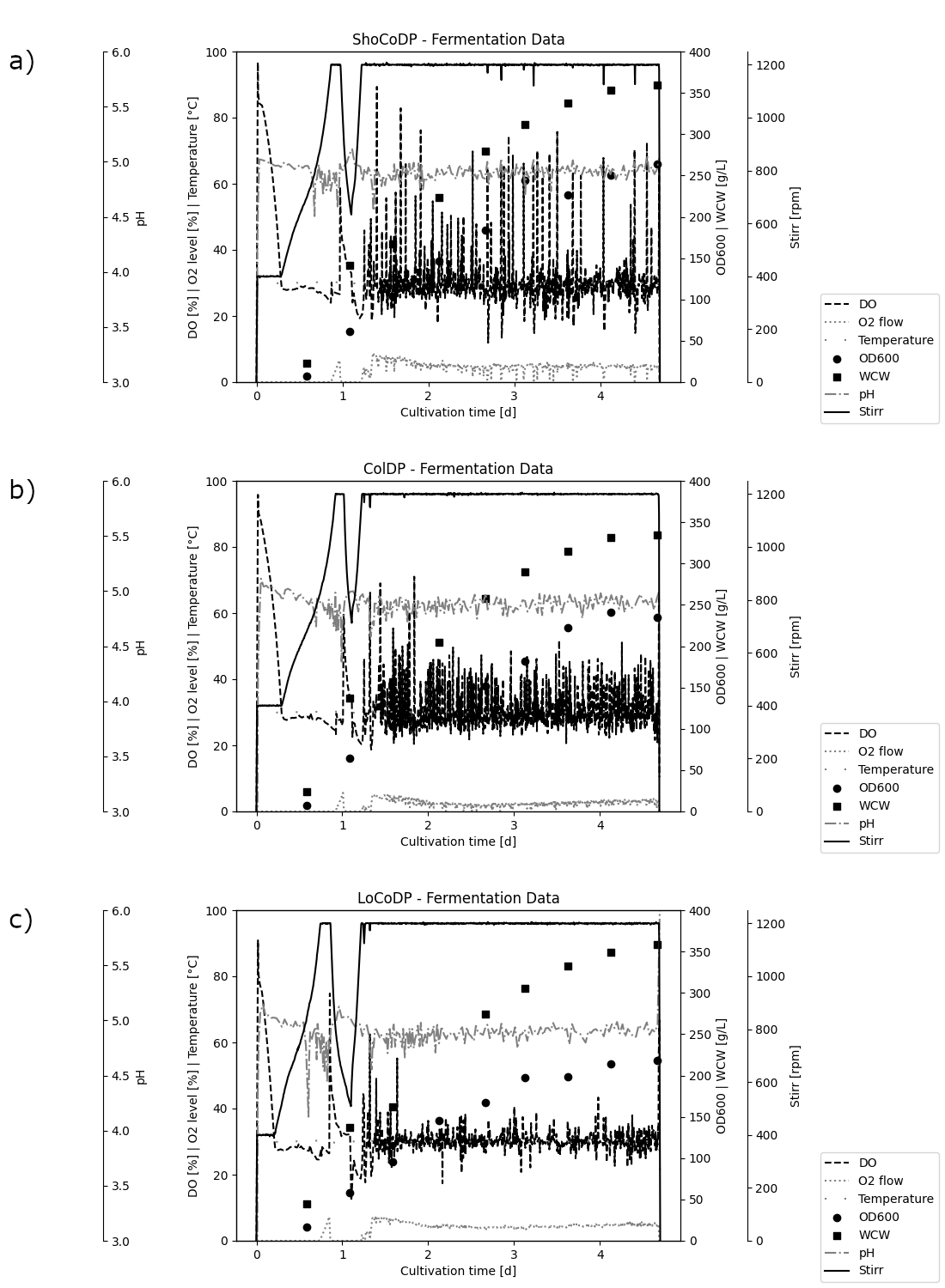


Figure S 1 Inline sensor data, wet cell weight and optical densities at 600 nm of the fermentations conducted for expression of a) ShoCoDP, b) ColDP and c) LoCoDP.

### DoE parameters and measured results

Table S 1. Parameters and observed results for the DoE performed for hydrogel characterization

| Name | Molecular weight [kDa] | MAA/g Protein [mL/g] | DoF  [%] | Reacive groups introduced  [mM/g] | Storage Modulus [Pa] | Deformation before breaking  [%] | Swelling  ratio |
| --- | --- | --- | --- | --- | --- | --- | --- |
| ShoCo Low | 25.6 | 0.035 | 60 | 0.431 | 1649 | 407 | 152.29 |
| ShoCo High | 25.6 | 0.1 | 77.66 | 0.559 | 3379 | 302 | 85.03 |
| LoCo Low | 89.2 | 0.03 | 41.98 | 0.284 | 3435 | 368 | 87.02 |
| LoCo High | 89.2 | 0.1 | 87.52 | 0.593 | 15640 | 167 | 34.77 |
| Col Mid 1 | 58 | 0.6 | 81.8 | 0.523 | 11630 | 203 | 39.4 |
| Col Mid 2 | 58 | 0.45 | 58.03 | 0.370 | 6368 | 273 | 67.77 |
| Col Mid 3 | 58 | 0.45 | 58 | 0.372 | 5386 | 287 | 68.56 |
| ShoCo Low 2 | 25.6 | 0.03 | 31.72 | 0.228 | No curing | - | - |

### ^
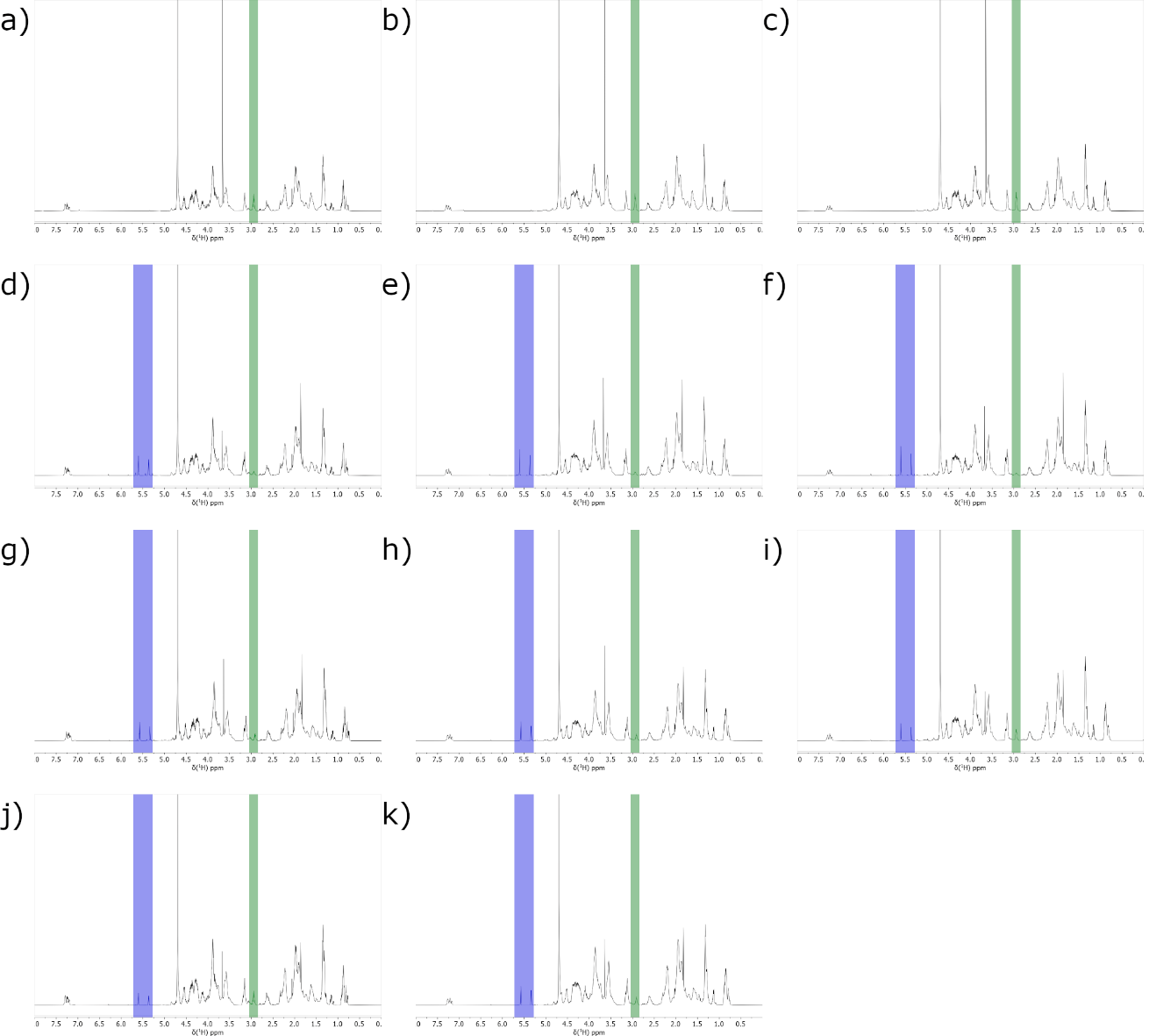
1^H-NMR spectra of unmodified and methactylated collagen derived proteins

Figure S 2 ^1^H NMR spectra of a) ShoCoDP, b) ColDP, c) LoCoDP, d) ShoCoDP-MA modified with 0.1 mL methacrylic anhydride per gram of protein, e) ColDP-MA modified with 0.06 mL methacrylic anhydride per gram of protein, f) LoCoDP-MA modified with 0.1 mL methacrylic anhydride per gram of protein, g) ShoCoDP-MA modified with 0.035 mL methacrylic anhydride per gram of protein, h)&k) ColDP-MA modified with 0.045 mL methacrylic anhydride per gram of protein, i) LoCoDP-MA modified with 0.03 mL methacrylic anhydride per gram of protein j) ShoCoDP-MA modified with 0.03 mL methacrylic anhydride per gram of protein. Peaks linked to the primary amine in the lysine side chain are marked in green, peaks attributed to the introduced methacryloyl groups are marked in purple.

### Quality control plots for the models fitted for DoE evaluation


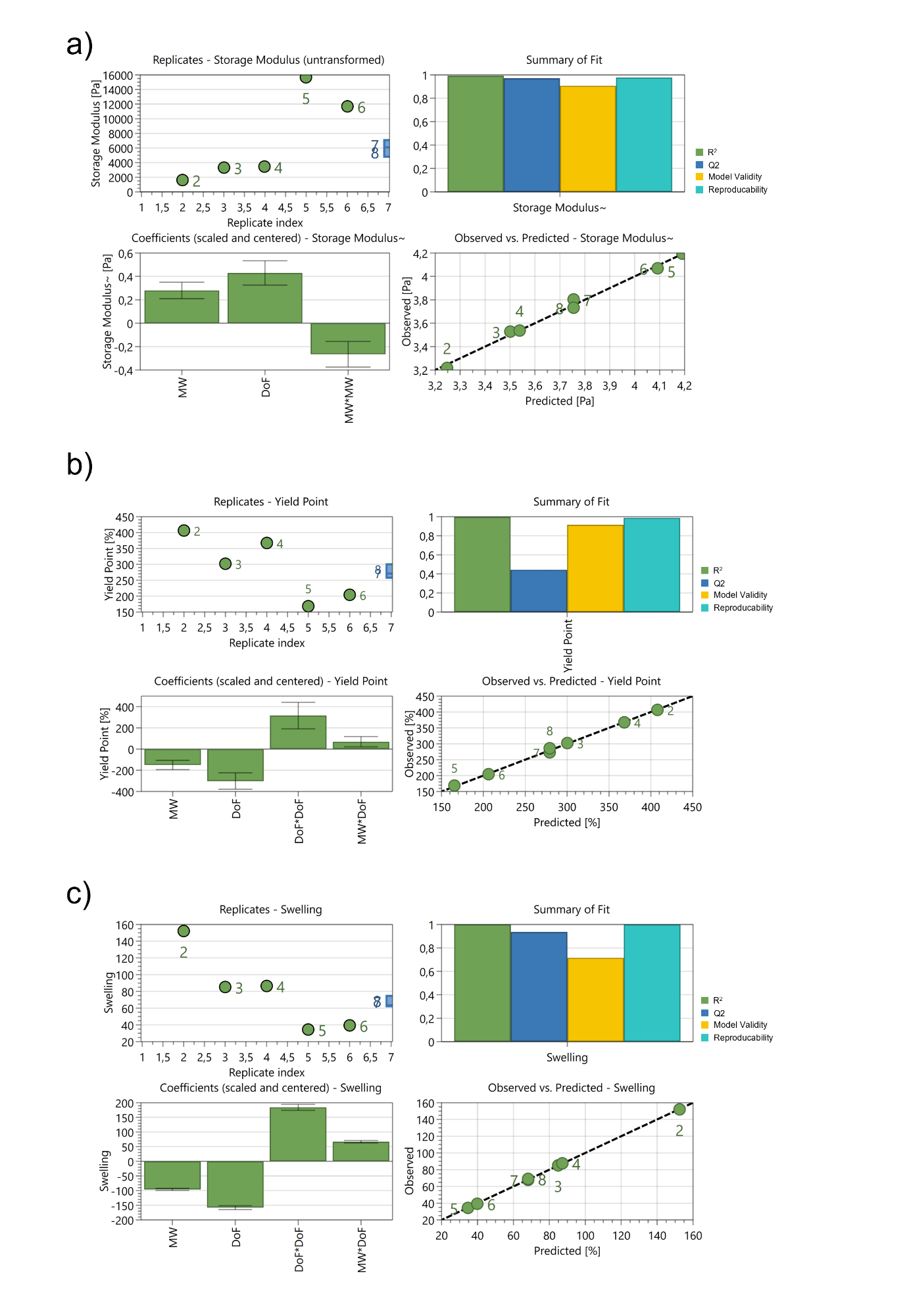


Figure S 3 Overview of the fitted models’ parameters for a) hydrogel stiffness, b) yield point and c) mass swelling ration of hydrogels prepared form collagen-derived proteins with different DoF and molecular weight.

### Stiffness of hydrogels prepared by mixtures if different DoF and molecular weight


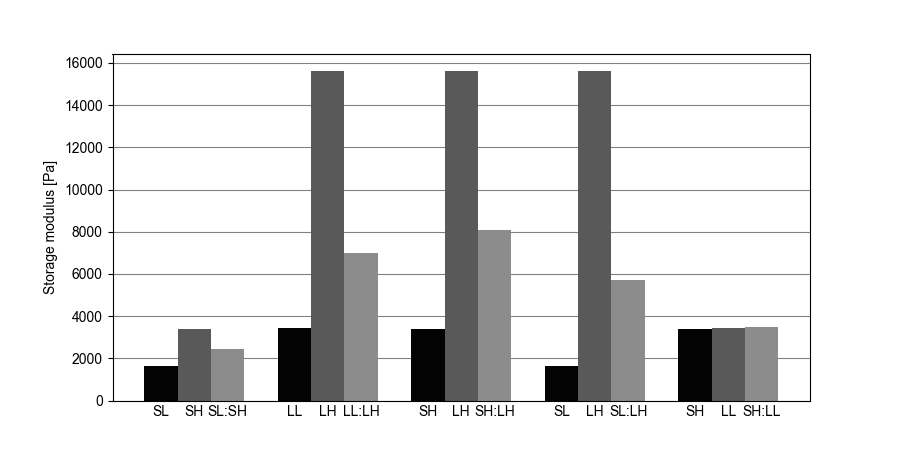


Figure S 4 Storage modulus of recombinant collagen derived proteins with varying DoF. (SL = ShoCoDP-MA Low, SH = ShoCoDP-MA High, LL = LoCoDP-MA Low, LH = LoCoDP-MA-High, SL:SH = 50:50 mixture of SL and SH, LL:LH = 50:50 mixture of LL and LH, SH:LH = 50:50.

### Protein sequences of the different collagen derived proteins used in this study

#### Short Collagen-Derived Protein (ShoCoDP)

EFTGSPGSPGPDGKTGPPGPAGQDGRPGPPGPPGARGQAGPPGFPGPKGAAGEPGKAGERGVPGPPGAVGPAGKDGEAGAQGPPGPAGPAGERGEQGPAGSPGFQGLPGPAGPPGEAGKPGEQGVPGDLGAPGPSGARGERGFPGERGVQGPPGPAGPRGANGAPGNDGAKGDAGAPGAPGSQGAPGLQGMPGERGAAGLPGPKGDRGDAGPKGADGSPGKDGVRGLTGPIGPPGPAGAPGDKGESGPSGPAGPTGARGAPGDRGEPGPPGPAGFAGPPGADGQPGAK

(Cell adhesive RGD sites are highlighted in yellow)

#### Collagen-Derived Protein (ColDP)

EFTGSPGSPGPDGKTGPPGPAGQDGRPGPPGPPGARGQAGPPGFPGPKGAAGEPGKAGERGVPGPPGAVGPAGKDGEAGAQGPPGPAGPAGERGEQGPAGSPGFQGLPGPAGPPGEAGKPGEQGVPGDLGAPGPSGARGERGFPGERGVQGPPGPAGPRGANGAPGNDGAKGDAGAPGAPGSQGAPGLQGMPGERGAAGLPGPKGDRGDAGPKGADGSPGKDGVRGLTGPIGPPGPAGAPGDKGESGPSGPAGPTGARGAPGDRGEPGPPGPAGFAGPPGADGQPGAKGEPGDAGAKGDAGPPGPAGPAGPPGPIGNVGAPGAKGARGSAGPPGATGFPGAAGRVGPPGPSGNAGPPGPPGPAGKEGGKGPRGETGPAGRPGEVGPPGPPGPAGEKGSPGADGPAGAPGTPGPQGIAGQRGVVGLPGQRGERGFPGLPGPSGEPGKQGPSGASGERGPPGPMGPPGLAGPPGESGREGAPGAEGSPGRDGSPGAKGDRGETGPAGPPGAPGAPGAPGPVGPAGKSGDRGETGPAGPTGPVGPVGARGPAGPQGPRGDKGETGEQGDRGIKGHRGFSGLQGPPGPPGSPGEQGPSGASGPAGPRGPPGSAGAPGKDGLNGLPGPIGPPGPRGRTGDAGPVGPPGPPGPPGPPGPP

(Cell adhesive RGD sites are highlighted in yellow)

#### Long Collagen-Derived Protein (LoCoDP)

EFTGSPGSPGPDGKTGPPGPAGQDGRPGPPGPPGARGQAGPPGFPGPKGAAGEPGKAGERGVPGPPGAVGPAGKDGEAGAQGPPGPAGPAGERGEQGPAGSPGFQGLPGPAGPPGEAGKPGEQGVPGDLGAPGPSGARGERGFPGERGVQGPPGPAGPRGANGAPGNDGAKGDAGAPGAPGSQGAPGLQGMPGERGAAGLPGPKGDRGDAGPKGADGSPGKDGVRGLTGPIGPPGPAGAPGDKGESGPSGPAGPTGARGAPGDRGEPGPPGPAGFAGPPGADGQPGAKGEPGDAGAKGDAGPPGPAGPAGPPGPIGNVGAPGAKGARGSAGPPGATGFPGAAGRVGPPGPSGNAGPPGPPGPAGKEGGKGPRGETGPAGRPGEVGPPGPPGPAGEKGSPGADGPAGAPGTPGPQGIAGQRGVVGLPGQRGERGFPGLPGPSGEPGKQGPSGASGERGPPGPMGPPGLAGPPGESGREGAPGAEGSPGRDGSPGAKGDRGETGPAGPPGAPGAPGAPGPVGPAGKSGDRGETGPAGPTGPVGPVGARGPAGPQGPRGDKGETGEQGDRGIKGHRGFSGLQGPPGPPGSPGEQGPSGASGPAGPRGPPGSAGAPGKDGLNGLPGPIGPPGPRGRTGDAGPVGPPGPPGPPGPPGPPKGDRGDAGPKGADGSPGKDGVRGLTGPIGPPGPAGAPGDKGESGPSGPAGPTGARGAPGDRGEPGPPGPAGFAGPPGADGQPGAKGEPGDAGAKGDAGPPGPAGPAGPPGPIGNVGAPGAKGARGSAGPPGATGFPGAAGRVGPPGPSGNAGPPGPPGPAGKEGGKGPRGETGPAGRPGEVGPPGPPGPAGEKGSPGADGPAGAPGTPGPQGIAGQRGVVGLPGQRGERGFPGLPGPSGEPGKQGPSGASGERGPPGPMGPPGLAGPPGESGREGAPGAEGSPGRDGSPGAKGDRGETGPAGPPGAPGAPGAPGPVGPAGKSGDRGETGPAGPTGPVGPVGARGPAGPQGPRGDK

(Cell adhesive RGD sites are highlighted in yellow)

1. [↑](#footnote-ref-1)
